# Why public health people are more worried than excited over e-cigarettes

**DOI:** 10.1186/s12916-014-0226-y

**Published:** 2014-12-09

**Authors:** Charlotta Pisinger

**Affiliations:** Research Centre for Prevention and Health, Glostrup Hospital, Building 84/85, Nordre Ringvej 57, DK 2600 Glostrup, Denmark

**Keywords:** Electronic cigarettes, ENDS, Harm reduction, Public health, Smoking, Vaping

## Abstract

The research field on e-cigarettes is characterized by severe methodological problems, severe conflicts of interest, relatively few and often small studies, inconsistencies and contradictions in results, and a lack of long-term follow-up. Therefore, no firm conclusions can be drawn on the harm of e-cigarettes, but they can hardly be called safe. Experimental studies indicate negative health effects and, amongst others, the major ingredient propylene glycol warrants concern. Growing evidence raises doubt about the efficacy of e-cigarettes as a smoking cessation aid. Unfortunately, it seems that many smokers use e-cigarettes with the intention to quit but switch to long-term use of e-cigarettes or dual use. Use is spreading rapidly to minors, ex-smokers, and never-smokers. It is questionable whether the potential health benefits obtained by some smokers outweigh the potential harm by use of non-smokers, of undermining of complete cessation, smokers’ dual use, and of eventual re-normalization of smoking. Even if e-cigarettes are significantly less harmful than conventional cigarettes, the product may have a very negative impact on public health if its use is spread to a large part of the population.

## Background

When I first heard about the e-cigarette (EC), I was excited. Was this the miraculous alternative to conventional cigarettes (CC) that could prevent millions of peoples’ suffering? Today, big tobacco companies have bought up the market, sales are exploding, and I and many other health professionals are worried [1].

Some harm reduction advocates claim that public health professionals are just moralists with an aversion of nicotine, an addictive drug, leading to an illogical and unfair hatred of ECs. I believe this subject is of too large public health importance to resort to mudslinging.

The harm reduction strategy (replacing a very harmful product with a less harmful product) is common sense; however, history has unfortunately shown that common sense can do harm [2]. As a doctor, I have sworn “First Do No Harm”, as a researcher, I call for substantial evidence with very consistent findings, and as a public health professional, I am obliged to take long-term consequences for the whole population – both smokers and non-smokers – into account before recommending a new product.

### The safety of e-cigarettes

ECs are marketed as safe products delivering pure nicotine and releasing harmless water vapor that vanishes in seconds [3,4], but is this true? The research field is characterized by severe methodological problems, severe conflicts of interest, relatively few and often small studies, inconsistencies and contradictions in results, and a lack of long-term follow-up. Therefore, no firm conclusions can be drawn on the safety of ECs [5,6], and much is left to subjective interpretations. Most probably ECs are less harmful than conventional cigarettes, but they can hardly be called safe. An experimental study found that cells exposed to high-nicotine vapor showed a similar pattern of gene expression to those exposed to tobacco smoke [7]. Very short-term experimental exposure to EC vapor showed effects that are reminiscent of the obstructive effects seen with smoking [8-10], even though the impact on lung function was smaller than with smoking. An experimental animal study found that EC fluid can exacerbate allergy-induced asthma symptoms [11]. Furthermore, a study found that the vapor induced release of cytokines and pro-inflammatory mediators [12] and many studies have found cytotoxicity [13,14] and harmful substances in fluid and vapor (e.g., fine or ultrafine particles [15-17], harmful metals [13,18], carcinogenic tobacco-specific nitrosamines [19-23], carbonyls [19,21,24,25], volatile organic compounds [19,26], polycyclic aromatic hydrocarbons [18,22]) or in urine [18]. It is true that most studies found low or very low concentrations, but values below the threshold-limit do not necessarily protect against a negative health effect of 200 to 300 daily inhalations [27] over decades.

The EC is a radically different product than the CC and, therefore, it seems wrong to base an assessment of the safety of EC on comparisons with CCs only. Of special concern are compounds not found in CCs: the glycols (propylene glycol and sometimes glycerin) are major ingredients of ECs [28-32] used to create the visible fume. A report commissioned by vapers and vendors of ECs concluded that *“estimated levels of exposure to propylene glycol and glycerin are close enough to threshold-limit values to warrant concern”* and that *“the threshold-limit value is based on uncertainty rather than knowledge”* [33]. Several studies on glycols have raised health concerns [34-37]. Other concerns are the flavors, metals, and silicone [13,14,38]. Finally, nicotine itself is probably not harmless [39], and it is highly addictive. Studies show that non-smokers passively exposed to ECs absorb nicotine [18,40,41].

### Use of e-cigarettes is no longer restricted to smokers

In the first years, the ECs were bought by smokers only; however, recently, their use has also spread to ex-smokers [42-45] and never smokers [45-49]. The intensive marketing, the novelty, the use of celebrities as role models, and the candy-like flavors appeal to young people. Experimental use in minors has doubled within one year [45,48,50]. A high proportion of adolescents have tried ECs [46,47,49], even at age 12 to 14 years [47]. Of special concern is that young never-smokers are experimenting with ECs [45-49] and the use of ECs might undermine decades of efforts to de-normalize smoking [51]. A brand new study from Poland finds that almost every third adolescent is current user of ECs and more than every fifth has a dual use of CCs and ECs. However, the most alarming finding is that the prevalence of smoking increased with increasing rates of EC use, from 24% to 38% during a period of only three years, indicating a renormalization of smoking [52].

It would be naïve not to expect that the manufacturers will try hard to spread the use of their product to as many consumers as possible, and history has shown that the tobacco industry has no ethical constraints [53].

### The effectiveness of the e-cigarette as an aid for smoking cessation

Making strong conclusions based on smoking rates and rates of EC-use in different countries is difficult, as smoking rates are influenced by political decisions as price and availability, changes in the social norm, etc. Some prospective studies were very promising [54,55], and a recent large ‘real-world’ study taking smoker’s addiction into account showed that use of ECs increased cessation rates more than no aid/nicotine replacement products bought over the counter [56]. However, a meta-analysis based on five population-based studies found that EC users were significantly less likely than non-users to have stopped smoking [6], a longitudinal study in cancer patients showed that EC users were twice as likely to be smoking at the time of follow-up as non-users [57], and the only existing randomized smoking cessation study showed that ECs were not significantly more effective than nicotine patch therapy [58]. A survey sponsored by EC manufacturers found that only 1% of EC users achieved permanent abstinence [59], but I have not seen this study cited by harm reduction advocates. Unfortunately, it seems that many smokers use ECs with the intention to quit but switch to long-term use of ECs [58] or end with dual use, supplementing their smoking with the EC [42-44,60] – dream-scenarios for the industry.

### Impact on public health

**Figure 1:**
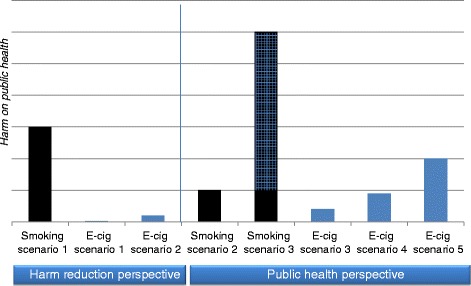
**The long-term impact of smoking and e-cigarette use on public health – year 2050.** The risk models are based on assumptions of prevalence of smoking and prevalence of use and harm of electronic cigarettes (EC). Harm of smoking is known to be extremely high; this is our reference. Maximal harm = 100. In a harm reduction perspective the harm of EC-use is estimated as extremely low/very low = 1 or 5. In a public health perspective the harm of EC-use is estimated low/moderate = 10, 15 or 25. ***Harm reduction perspective:*** *Smoking scenario 1*: the theory assumes that smokers are reluctant to quit and smoking rates will remain high (15% smokers, harm =100). **E-cigarette scenarios**: the theory assumes that harm of EC-use is extremely low/very low and use will be restricted to smokers only. *EC-scenario 1*: 10% of the population use ECs, harm = 1. *EC-scenario 2 (worst case)*: 20% of the population (primarily smokers) use ECs, harm = 5. ***Public health perspective:*** *Smoking scenario 2*: the theory is that smokers wish to quit and tobacco control efforts are effective. Smoking rates will reduce steadily over the next decades (5% smokers, harm =100). *Smoking scenario 3 (worst case)*: EC-use might undermine smoking cessation and renormalize conventional smoking, and the smoking rates might increase. The harm indicated as squared is the extra harm indirectly caused by ECs (30% smokers, harm = 100). **E-cigarette scenarios**: according to the theory we might underestimate long-term harm of ECs, and use of ECs might spread to a large part of the population *EC-scenario 3*: 20% of the population use ECs, harm = 10. *EC-scenario 4*: 30% of the population use ECs, harm = 15. *EC-scenario 5 (worst case)*: 40% of the population use ECs, harm = 25.

**Figure 2:**
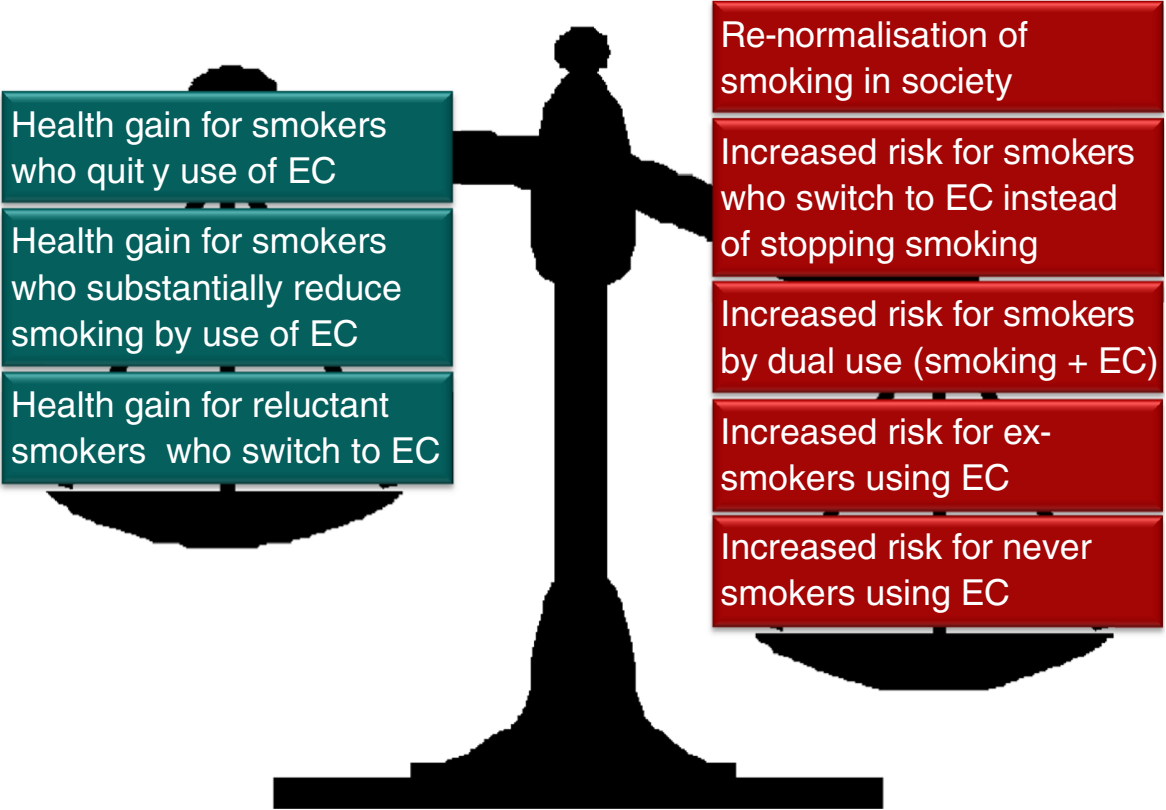
**The difficult balance between the potential pros and cons of e-cigarettes.** The public health perspective.

When we compare with a CC, the most harmful legal product on the market, everything seems harmless. For a smoker reluctant to stop smoking, the EC will most probably be a less harmful alternative – but we cannot focus on these smokers only! The impact of a product on public health is determined by two factors: i) the degree of toxicity or harm of the substance; and ii) how widespread the exposure is. Even if ECs are significantly less harmful than CCs, the product may have a negative impact on public health if the use is spread to a large part of the population (Figure 1). ECs might achieve popularity as high as that of CCs in the 1960s, before an awareness of harm became widespread in the population. The potential health benefits obtained by some smokers (Figure 2) must outweigh the potential harm by use of ex- and never-smokers, of smokers who intended to quit but switched to ECs, of smokers’ dual use, and of eventual re-normalization of smoking.

## Conclusions

Most probably, ECs are less harmful than CCs, but they can hardly be called safe. Consequences of EC use must be viewed in a long-term public health perspective, including both smokers and non-smokers. Based on the limited and often conflicting evidence on safety, the doubtful efficacy as a smoking cessation aid, and the alarming rise in use in young people and non-smokers, most public health professionals urge great caution with ECs and call for regulation, monitoring, and research not biased by conflicts of interest.

As the WHO states, this is an *“evolving frontier filled with promise and threat for tobacco control”* [1]. I shall be the first to celebrate if the ECs turn out to be a safe and effective weapon in tobacco control. Till then, let us focus on intensifying our fight for a smoke-free world by restricting the influence of the tobacco industry, by regulating smoking and other tobacco-/nicotine containing products, and by offering evidence-based help for smoking cessation – we know this works.

## Abbreviations

CC: Conventional cigarette
EC: Electronic cigarette
